# Supplementary material for: The Cost-Effectiveness of the Human Papilloma Virus Vaccination in Asia Pacific Countries: What Lessons Can Indonesia Learn?—A Systematic Review
Source: Vaccines (Basel). 2025 May 30;13(6):593. doi: 10.3390/vaccines13060593 (PMC12197709; doi:10.3390/vaccines13060593)
Supplement: Supplementary file 1 [file vaccines-13-00593-s001.zip › vaccines-3650034-supplementary.pdf]

Supplementary Materials

Table S1. PRISMA 2020 Checklist

Section and Topic	Item #	Checklist item	Location where item is reported
**TITLE**			
Title	1	Identify the report as a systematic review.	Title page, page 1
**ABSTRACT**			
Abstract	2	Provided a structured summary including, as applicable: background; objectives; data sources; study eligibility criteria; study appraisal and synthesis methods; results; conclusions and implications of key findings	Abstract, page 1
**INTRODUCTION**			
Rationale	3	Describe the rationale for the review in the context of existing knowledge.	Page 1-3
Objectives	4	Provide an explicit statement of the objective(s) or question(s) the review addresses.	Page 3
**METHODS**			
Eligibility criteria	5	Specify the inclusion and exclusion criteria for the review and how studies were grouped for the syntheses.	Page 3
Information sources	6	Specify all databases, registers, websites, organisations, reference lists and other sources searched or consulted to identify studies. Specify the date when each source was last searched or consulted.	Page 3
Search strategy	7	Present the full search strategies for all databases, registers and websites, including any filters and limits used.	Page 3 and Supplementary Table S1
Selection process	8	Specify the methods used to decide whether a study met the inclusion criteria of the review, including how many reviewers screened each record and each report retrieved, whether they worked independently, and if applicable, details of automation tools used in the process.	Page 3 & 4
Data collection process	9	Specify the methods used to collect data from reports, including how many reviewers collected data from each report, whether they worked independently, any processes for obtaining or confirming data from study investigators, and if applicable, details of automation tools used in the process.	Page 3 & 4
Data items	10a	List and define all outcomes for which data were sought. Specify whether all results that were compatible with each outcome domain in each study were sought (e.g. for all measures, time points, analyses), and if not, the methods used to decide which results to collect.	Page 3 & Supplementary Table S2-S5
	10b	List and define all other variables for which data were sought (e.g. participant and intervention characteristics, funding sources). Describe any assumptions made about any missing or unclear information.	Page 3 & Supplementary Table S3-S5
Study risk of bias assessment	11	Specify the methods used to assess risk of bias in the included studies, including details of the tool(s) used, how many reviewers assessed each study and whether they worked independently, and if applicable, details of automation tools used in the process.	Page 3 & 4 & Supplementary

Section and Topic	Item #	Checklist item	Location where item is reported
			Table S2
Effect measures	12	Specify for each outcome the effect measure(s) (e.g. risk ratio, mean difference) used in the synthesis or presentation of results.	NA
Synthesis methods	13a	Describe the processes used to decide which studies were eligible for each synthesis (e.g. tabulating the study intervention characteristics and comparing against the planned groups for each synthesis (item #5)).	Page 4
	13b	Describe any methods required to prepare the data for presentation or synthesis, such as handling of missing summary statistics, or data conversions.	Page 4
	13c	Describe any methods used to tabulate or visually display results of individual studies and syntheses.	Page 4
	13d	Describe any methods used to synthesize results and provide a rationale for the choice(s). If meta-analysis was performed, describe the model(s), method(s) to identify the presence and extent of statistical heterogeneity, and software package(s) used.	Page 4
	13e	Describe any methods used to explore possible causes of heterogeneity among study results (e.g. subgroup analysis, meta-regression).	NA
	13f	Describe any sensitivity analyses conducted to assess robustness of the synthesized results.	NA
Reporting bias assessment	14	Describe any methods used to assess risk of bias due to missing results in a synthesis (arising from reporting biases).	NA
Certainty assessment	15	Describe any methods used to assess certainty (or confidence) in the body of evidence for an outcome.	NA
**RESULTS**			
Study selection	16a	Describe the results of the search and selection process, from the number of records identified in the search to the number of studies included in the review, ideally using a flow diagram.	Page 4 & Figure 1
	16b	Cite studies that might appear to meet the inclusion criteria, but which were excluded, and explain why they were excluded.	Figure 1
Study characteristics	17	Cite each included study and present its characteristics.	Page 4-5 & Table 1 & Figure 2 & Supplementary Table S3-S6
Risk of bias in studies	18	Present assessments of risk of bias for each included study.	NA
Results of individual studies	19	For all outcomes, present, for each study: (a) summary statistics for each group (where appropriate) and (b) an effect estimate and its precision (e.g. confidence/credible interval), ideally using structured tables or plots.	Page 4-10 & Table 1 & Figure 2-3 & Supplementary Table S3-S6
Results of	20a	For each synthesis, briefly summarise the characteristics and risk of bias among contributing studies.	Page 4-10 & Table 1 &

Section and Topic	Item #	Checklist item	Location where item is reported
syntheses			Figure 2 & Supplementary Table S3-S6
	20b	Present results of all statistical syntheses conducted. If meta-analysis was done, present for each the summary estimate and its precision (e.g. confidence/credible interval) and measures of statistical heterogeneity. If comparing groups, describe the direction of the effect.	Page 5-8 & Table 1 & Figure 2 & Supplementary Table S3
	20c	Present results of all investigations of possible causes of heterogeneity among study results.	NA
	20d	Present results of all sensitivity analyses conducted to assess the robustness of the synthesized results.	NA
Reporting biases	21	Present assessments of risk of bias due to missing results (arising from reporting biases) for each synthesis assessed.	NA
Certainty of evidence	22	Present assessments of certainty (or confidence) in the body of evidence for each outcome assessed.	NA
**DISCUSSION**			
Discussion	23a	Provide a general interpretation of the results in the context of other evidence.	Page 10-11
	23b	Discuss any limitations of the evidence included in the review.	Page 10-11
	23c	Discuss any limitations of the review processes used.	Page 11
	23d	Discuss implications of the results for practice, policy, and future research.	Page 11
**OTHER INFORMATION**			
Registration and protocol	24a	Provide registration information for the review, including register name and registration number, or state that the review was not registered.	Page 3
	24b	Indicate where the review protocol can be accessed, or state that a protocol was not prepared.	NA
	24c	Describe and explain any amendments to information provided at registration or in the protocol.	NA
Support	25	Describe sources of financial or non-financial support for the review, and the role of the funders or sponsors in the review.	Page 12
Competing interests	26	Declare any competing interests of review authors.	Page 12
Availability of data, code and other materials	27	Report which of the following are publicly available and where they can be found: template data collection forms; data extracted from included studies; data used for all analyses; analytic code; any other materials used in the review.	Page 12

From: Page MJ, McKenzie JE, Bossuyt PM, Boutron I, Hoffmann TC, Mulrow CD, et al. The PRISMA 2020 statement: an updated guideline for reporting systematic reviews. BMJ 2021;372:n71. doi: 10.1136/bmj.n71. This work is licensed under CC BY 4.0. To view a copy of this license, visit https://creativecommons.org/licenses/by/4.0/

Table S2. Search strings used for the systematic review

#1 Primary Prevention	'primary prevention'/exp OR 'primary prevention' OR 'immunization'/exp OR 'immunization' OR 'vaccination policy'/exp OR 'vaccination policy' OR 'vaccination program'/exp OR 'vaccination program' OR 'vaccination programme'/exp OR 'vaccination programme' OR 'virus vaccination'/exp OR 'virus vaccination' OR 'vaccination'/exp OR 'vaccination'
#2 HPV Vaccination	'ceravix' OR 'cervarix' OR 'gardasil' OR 'gardasil 9' OR 'human papillomavirus recombinant vaccine quadrivalent, types 6, 11, 16, 18' OR 'human papillomavirus type 16 vaccine' OR 'human papillomavirus type 18 vaccine' OR 'human papillomavirus vaccine*' OR 'human papillomavirus vaccine* (types 16, 18)' OR 'human papillomavirus vaccine (types 6, 11, 16, 18) , recombinant, adsorbed' OR 'papilloma virus vaccine*' OR 'papillomavirus vaccine*' OR 'papillomavirus vaccines' OR 'silgard' OR 'wart virus vaccine*' OR 'human papilloma virus vaccine*' OR 'hpv l1 vaccine, quadrivalent 6, 11, 16, 18' OR 'human papillomavirus vaccine l1, type* 6, 11, 16, 18' OR 'hpv vaccin*'
#3 Cervical Cancer	cancer of cervix' OR 'cancer of the cervix' OR 'cancer of the uterine cervix' OR 'cervical neoplasm*' OR 'uterine cervical cancer*' OR 'uterine cervical neoplasm*' OR 'ca cervix' OR 'cancer of the cervix uterine' OR 'cancer, uterine cervix' OR 'cervical cancer' OR 'cervical malignancies' OR 'cervical malignancy' OR 'cervix ca' OR 'cervix cancer' OR 'cervix cancer, uterine' OR 'cervix malignancies' OR 'cervix malignancy' OR 'cervix uteri cancer' OR 'cervix uterus cancer' OR 'malignancies of the cervix' OR 'malignancy of the cervix' OR 'uterine cervical cancer' OR 'uterine cervix malignancy, recurrent' OR 'uterine neck cancer' OR 'uterus cervix cancer' OR 'uterine cervix cancer'
#4 Selected Asia Pacific Countries	siam' OR 'thailand' OR 'kingdom of thailand' OR 'vietnam*' OR 'viet nam' OR 'vietnam, republic of' OR 'beijing' OR 'chinese people* republic' OR 'people* republic of china' OR 'china' OR 'inner mongolia' OR 'mainland china' OR 'manchuria' OR 'sinkiang' OR 'singapor*' OR 'malaysia*' OR 'federation of malaya' OR 'malay federation' OR 'malay peninsula' OR 'malaya' OR 'malaya federation' OR 'sabah' OR 'sarawak' OR 'the philippines' OR 'philippin*' OR 'philipines' OR 'phillipines' OR 'phillippines' OR 'korea*' OR 'democratic people* republic of korea' OR 'republic of korea' OR 'tokyo' OR 'japan*' OR 'bonin islands' OR 'formosa' OR 'nationalist china' OR 'taiwan*' OR 'republic of china' OR 'australia*' OR 'canton and enderbury islands' OR 'christmas island' OR 'new zealand*' OR 'hong kong special administrative region' OR 'hongkong' OR 'hong kong' OR 'kowloon' OR 'new territories'
#5	#1 OR #2
#6	#1 AND #2
#7	#3 AND #4 AND #5
#8	#3 AND #4 AND #6

Table S3. Quality of reporting according to CHEERS 2022 checklist[1]

	Title	Abstract	Introduction	Methods																		Results				Discussion	Other relevant information	
**Authors, years of publication**	**1**	**2**	**3**	**4**	**5**	**6**	**7**	**8**	**9**	**10**	**11**	**12**	**13**	**14**	**15**	**16**	**17**	**18**	**19**	**20**	**21**	**22**	**23**	**24**	**25**	**26**	**27**	**28**
Aljunid et al., 2016[2]	R	R	R	R	R	R	R	R	R	R	R	R	R	R	R	R	R	R	R	NA	R	R	R	NA	R	R	R	R
Blakely et al., 2014[3]	R	R	R	R	R	R	R	R	R	R	R	R	R	R	R	R	R	R	R	R	R	R	R	R	R	R	R	R
Cheung et al., 2021[4]	R	R	R	R	R	R	R	R	R	R	R	R	R	R	R	R	R	R	R	NA	R	R	R	NA	R	R	R	R
Cheung et al., 2023[5]	R	R	R	R	R	R	R	R	R	R	R	R	R	R	R	R	R	R	R	NA	R	R	R	NA	R	R	R	R
Chou et al., 2022[6]	R	R	R	R	R	R	R	NA	R	R	R	R	R	R	R	R	R	R	R	NA	R	R	R	NA	R	R	R	R
Cody et al., 2021[7]	R	R	R	R	R	R	R	R	R	R	R	R	R	R	R	R	R	R	R	R	R	R	R	R	R	R	NA	R
Connelly et al., 2015[8]	R	R	R	R	R	R	R	R	R	R	R	R	R	R	R	R	R	R	R	R	R	R	R	R	R	R	R	R
Dasbach et al., 2008[9]	R	R	R	R	R	R	R	R	R	R	R	R	R	R	R	R	R	R	R	R	R	R	R	R	R	R	NA	R
Demarteau et al., 2012[10]	R	R	R	R	R	R	R	R	R	R	R	R	R	R	R	R	R	R	R	R	R	R	R	R	R	R	R	R
Ezat et al., 2010[11]	R	R	R	R	R	R	R	R	R	R	R	R	R	R	R	R	R	R	R	NA	R	R	R	NA	R	R	R	NA
Ezat et al., 2010[12]	R	R	R	R	R	R	R	R	R	R	R	R	R	R	R	NA	R	R	R	NA	R	R	R	NA	R	R	NA	R
Germar et al., 2016[13]	R	R	R	R	R	R	R	R	R	R	R	R	R	R	R	R	R	R	R	NA	R	R	R	NA	R	R	R	R
Jiang et al., 2019[14]	R	R	R	R	R	R	R	R	R	R	R	R	R	R	R	R	R	R	R	NA	R	R	R	NA	R	R	NA	R
Kim et al., 2008[15]	R	R	R	R	R	R	R	R	NA	R	R	R	R	R	R	R	R	R	R	R	R	R	R	R	R	R	R	NA
Konno et al., 2010[16]	R	R	R	R	R	R	R	R	R	R	R	R	R	R	R	R	R	R	R	R	R	R	R	R	R	R	NA	NA
Kulasingam et al., 2007[17]	R	R	R	R	R	R	R	R	R	R	R	R	R	R	R	R	R	R	R	NA	R	R	R	NA	R	R	R	R
Lee et al., 2011[18]	R	R	R	R	R	R	R	R	R	R	R	R	R	R	R	R	R	R	R	R	R	R	R	R	R	R	R	R
Levin et al., 2015[19]	R	R	R	R	R	R	R	R	NA	NA	R	R	R	R	R	R	R	R	R	R	R	R	R	R	R	R	R	R
Liu PH et al., 2010[20]	R	R	R	R	R	R	R	R	R	R	R	R	R	R	R	R	R	R	R	R	R	R	R	R	R	R	R	R
Liu YJ et al., 2016[21]	R	R	R	R	R	R	R	R	R	R	R	R	R	R	R	R	R	R	R	R	R	R	R	R	R	R	R	R

Llave et al., 2022[22]	R	R	R	R	R	R	R	R	R	R	R	R	R	R	R	R	R	R	R	R	R	R	R	R	R	R	R	R
Luo et al., 2020[23]	R	R	R	R	R	R	R	R	R	R	R	R	R	R	R	R	R	R	R	R	R	R	R	R	R	R	R	R
Mahumud et al., 2019[24]	R	R	R	R	R	R	R	R	R	R	R	R	R	R	R	R	R	R	R	R	R	R	R	R	R	R	R	R
Ma et al., 2020[25]	R	R	R	R	R	R	R	R	R	R	R	R	R	R	R	R	R	R	R	R	R	R	R	R	R	R	R	R
Mo et al., 2017[26]	R	R	R	R	R	R	R	R	NA	R	R	R	R	R	R	R	R	R	R	R	R	R	R	R	R	R	R	R
Phua et al., 2021[27]	R	R	R	R	R	R	R	R	R	R	R	R	R	R	R	R	R	R	R	R	R	R	R	R	R	R	R	R
Sharma et al., 2012[28]	R	R	R	R	R	R	R	R	R	R	R	R	R	R	R	R	R	R	R	R	R	R	R	R	R	R	R	R
Sharma et al., 2015[29]	R	R	R	R	R	R	R	R	R	R	R	R	R	R	R	R	R	R	R	NA	R	R	R	NA	R	R	R	R
Simms et al., 2016[30]	R	R	R	R	R	R	R	R	R	R	R	R	R	R	R	R	R	R	R	R	R	R	R	R	R	R	R	R
Tang et al., 2019[31]	R	R	R	R	R	R	R	R	R	R	R	R	R	R	R	R	R	R	R	R	R	R	R	R	R	R	R	R
Tay et al., 2017[32]	R	R	R	R	R	R	R	R	R	R	R	R	R	R	R	R	R	R	R	NA	R	R	R	NA	R	R	R	R
Tay et al., 2017[33]	R	R	R	R	R	R	R	R	R	R	R	R	R	R	R	R	R	R	R	NA	R	R	R	NA	R	1	NA	R
Tay et al., 2018[34]	R	R	R	R	R	R	R	R	R	R	R	R	R	R	R	R	R	R	R	R	R	R	R	R	R	R	R	R
Termrungruanglert et al., 2012[35]	R	R	R	R	R	R	R	R	R	R	R	R	R	R	R	R	R	R	R	NA	R	R	R	NA	R	R	NA	R
Termrungruanglert et al., 2021[36]	R	R	R	R	R	R	R	R	R	R	R	R	R	R	R	R	R	R	R	NA	R	R	R	NA	R	R	R	R
Van Kriekinge et al., 2017[37]	R	R	R	R	R	R	R	R	R	R	R	R	R	R	R	R	R	R	R	R	R	R	R	R	R	R	R	R
Van Minh et al., 2017[38]	R	R	R	R	R	R	R	R	R	R	R	R	R	R	R	R	R	R	R	R	R	R	R	R	R	R	R	R
Wahab et al., 2023[39]	R	R	R	R	R	R	R	R	R	R	R	R	R	R	R	R	R	R	R	R	R	R	R	R	R	R	NA	R
Yamabe et al., 2014[40]	R	R	R	R	R	R	R	R	R	R	R	R	R	R	R	R	R	R	R	NA	R	R	R	NA	R	R	R	R
Yamamoto et al., 2011[41]	R	R	R	R	R	R	R	R	R	R	R	R	R	R	R	R	R	R	R	NA	R	R	R	NA	R	R	R	R
Zhang et al., 2016[42]	R	R	R	R	R	R	R	R	R	R	R	R	R	R	R	R	R	R	R	R	R	R	R	R	R	R	R	R
Zhou et al., 2022[43]	R	R	R	R	R	R	R	R	NA	R	R	R	R	R	R	R	R	R	R	R	R	R	R	R	R	R	R	R
Zou et al., 2020[44]	R	R	R	R	R	R	R	R	R	R	R	R	R	R	R	R	R	R	R	NA	R	R	R	NA	R	R	R	R

R= Reported; NA= Not available

Table S4. Characteristics of forty-three included studies

Characteristics	Number of studies (n)	Percentage (%)
Selected articles	43	100
**Year of publication**		
2007	1	2
2008	2	5
2010	5	12
2011	2	5
2012	2	5
2013	1	2
2014	1	2
2015	3	7
2016	5	12
2017	5	12
2018	1	2
2019	3	7
2020	3	7
2021	4	9
2022	3	7
2023	2	5
**Name of journal**		
BMC Public Health	3	7
Vaccine	6	14
BMC Cost Effectiveness and Resource Allocation	1	2
Human Vaccines & Immunotherapeutics	4	9
Value in Health Regional Issues	2	5
BMC Infectious Diseases	2	5
Sexual Health	2	5
Asian Pacific Journal of Cancer Prevention	5	12
Value in Health	2	5
BMJ Open	1	2
International Journal of Gynecological Cancer	1	2
BMC Health Services Research	2	5
BMC Cancer	2	5
Plos One	2	5
BJOG: An International Journal of Obstetrics & Gynecology	4	9
The Lancet Public Health	1	2
International Journal of Gynecology & Obstetrics	1	2
Singapore Medical Journal	1	2
The Lancet Global Health	1	2

Table S5. Study objectives included in the systematic review

Authors, years of publication	Country	Economic Classification	Study Objective
Aljunid et al., 2016[2]	Malaysia	UMIC	To estimate the economic burden of different dose regimes of HPV vaccinations in preventing cervical cancers.
Blakely et al., 2014[3]	New Zealand	HIC	To assess the health impact (QALY), cost (health system perspective) and cost-effectiveness for three interventions:<br>1) the 2008 'as implemented' HPV vaccination programme of girls only in New Zealand;<br>2) modification to 'as implemented' to be a school-only programme as per Australia;<br>3) A new mandatory law was added requiring active opting-out of vaccination.

Cheung et al., 2021[4]	Hong Kong	HIC	To assess the impact of a school-based strategy comprising two-dose 9vHPV routine vaccination of 12-year-old girls with or without catch-up 9vHPV vaccination among females aged 13-18 years (two-dose catch-up among those aged 13-14 and three-dose catch up among those aged ≥ 15 years) along with screening in the Hong Kong setting
Cheung et al., 2023[5]	Hong Kong	HIC	To assess the public health impact and cost-effectiveness of routine gender-neutral vaccination (GNV) compared with routine female-only vaccination (FOV) with the 9vHPV vaccination in Hong Kong.
Chou et al., 2022[6]	Taiwan	HIC	To estimate the epidemiologic and economic impact of a 9vHPV vaccination program for 13- to 14-year-old females compared with that of the 2vHPV vaccine in Taiwan.
Cody et al., 2021[7]	Japan	HIC	To assess the health impact and cost-effectiveness of routine and catch-up vaccination of girls and women aged 11-26 years with a 4vHPV or 9vHPV vaccine in Japan compared with no vaccination
Connelly et al., 2015[8]	Japan	HIC	To assess the cost-effectiveness of introducing a 2vHPV HPV vaccination program in Japan from a healthcare perspective
Dasbach et al., 2008[9]	Taiwan	HIC	To examine the potential long-term epidemiologic and economic consequences of a quadrivalent HPV (6/11/16/18) vaccination program in Taiwan
Demarteau et al., 2012[10]	Taiwan	HIC	To compare the epidemiological and economic impact of cross-protection against oncogenic HPV types beyond 16/18 of the 2vHPV vaccine versus protection against non-oncogenic HPV types 6/11 of the 4vHPV vaccine in Taiwan
Ezat et al., 2010[11]	Malaysia	UMIC	Cost-effectiveness options were compared for three programs, i.e. screening via Pap smear: modelling of HPV vaccination (QV and BV) and combined strategy (screening plus vaccination)
Ezat et al., 2010[12]	Malaysia	UMIC	To undertake a cost analysis of the management of cervical cancer cases in Malaysia by government healthcare providers and to estimate the economic burden of cervical cancer in the Malaysian population. Three alternative options were compared: 1) Pap smear screening, 2) 4vHPV vaccination, and 3) combined screening plus vaccination.
Germar et al., 2016[13]	Philippines	LMIC	To evaluate the cost-effectiveness of HPV vaccination of 13-year-old Filipino girls, in addition to current screening, in the new 2D schedule.
Jiang et al., 2019[14]	China	UMIC	To analyse the cost-effectiveness of the 9-valent HPV vaccine for the prevention of cervical cancer among Chinese women
Kim et al., 2008[15]	Vietnam	LMIC	To explore and quantify the clinical and economic trade-offs of establishing a national versus region-based policy for cervical cancer prevention
Konno et al., 2010[16]	Japan	HIC	To quantify the clinical impact and assess the cost-effectiveness of adding CC vaccination at age 12 to the current screening in place in Japan.
Kulasingam et al., 2007[17]	Australia	HIC	To examine the cost-effectiveness of adding an HPV vaccine to the Australian National Cervical Cancer Screening Program compared to screening alone
Lee et al., 2011[18]	Singapore	HIC	To explore the cost-effectiveness of 2vHPV vaccine and 4vHPV vaccine in Singapore
Levin et al., 2015[19]	China	UMIC	To evaluate public financing of HPV vaccination to prevent cervical cancer, adding new dimensions to conventional cost-effectiveness analysis through explicit inclusion of equity and impact on financial risk protection.
Liu PH et al., 2010[20]	Taiwan	HIC	To assess the cost-effectiveness of prophylactic HPV vaccination for the prevention of cervical cancer in Taiwan.
Liu YJ et al., 2016[21]	China	UMIC	To estimate the cost-effectiveness of a 3-dose 2vHPV HPV vaccination at ages 12 to 55 years in both rural and urban settings in China.
Llave et al., 2022[22]	Philippines	LMIC	To estimate the potential cost-effectiveness of four different HPV vaccine products—Cervarix, Cecolin, GARDASIL, and GARDASIL 9—for routine HPV vaccination of 10 cohorts of 9-year-old girls from the government and societal perspectives.

Luo et al., 2020[23]	China	UMIC	To assess the incremental cost-effectiveness of two doses of human papillomavirus (HPV) vaccination (bivalent 16/18 vaccine; 2vHPV) compared to a no-vaccination scenario and a three-dose scenario in one province in China.
Mahumud et al., 2019[24]	Australia	HIC	The cost-effectiveness of controlling cervical cancer using a new 9-valent human papillomavirus vaccine among school-aged girls in Australia
Ma et al., 2020[25]	China	UMIC	To evaluate the population impact and cost-effectiveness of strategies that combined cervical cancer screening and HPV school girl vaccination for Chinese women
Mo et al., 2017[26]	China	UMIC	To assess the cost-effectiveness of 2-, 4-, and 9-valent HPV vaccines (hereafter, HPV2, 4 or 9) combined with current screening strategies in China.
Phua et al., 2021[27]	Singapore	HIC	To evaluate the cost-effectiveness of replacing the bivalent vaccine with the nonavalent vaccine in the national school-based HPV vaccination programme in Singapore
Sharma et al., 2012[28]	Thailand	UMIC	To assess the health and economic outcomes of various screening and vaccination strategies for cervical cancer prevention
Sharma et al., 2015[29]	Vietnam	LMIC	To estimate the health benefits and incremental cost-effectiveness of human papillomavirus (HPV) vaccination of preadolescent boys and girls compared with girls alone for preventing cervical cancer and genital warts.
Simms et al., 2016[30]	Australia	HIC	To evaluate the cost-effectiveness of HPV9 in Australia, a country with HPV vaccination of both sexes that is transitioning To 5-yearly HPV-based screening.
Tang et al., 2019[31]	Taiwan	HIC	To assess the cost-effectiveness of vaccination with 2D-AS04-HPV-16/18v combined with screening, compared with (i) screening alone, (ii) 2D-4vHPVv combined with screening, (iii) 2D-9vHPVv combined with screening; (iv) 3D-9vHPVv combined with screening.
Tay et al., 2017[32]	Singapore	HIC	To examine the epidemiological and economic impact of a nine-valent (nonavalent) human papillomavirus (HPV) 6/11/16/18/31/33/45/52/58 vaccine programme for young teenagers in Singapore.
Tay et al., 2017[33]	Singapore	HIC	To investigate the clinical and economic impacts of school-based administration of the quadrivalent HPV vaccine. 2 strategies: 1) only girls, with a 5-year catch-up vaccination among those aged 13-17 years. 2) both girls and boys with no catch-up vaccination
Tay et al., 2018[34]	Singapore	HIC	To evaluate the cost-effectiveness of two-dose HPV vaccination as part of a national vaccination programme for 12-year-old girls in Singapore from the healthcare payer's perspective.
Termrungruanglert et al., 2012[35]	Thailand	UMIC	To simulate the lifetime economic impact for women in the context of human papillomavirus (HPV) infection prevention
Termrungruanglert et al., 2021[36]	Thailand	UMIC	To examine the epidemiological consequences and cost-effectiveness of a routine quadrivalent HPV (4vHPV) vaccination and the routine 4vHPV vaccination plus 5-year catch-up vaccination by comparing with cervical cancer screening only (no vaccination) in Thailand.
Van Kriekinge et al., 2017[37]	Malaysia	UMIC	To comparatively evaluate the results of a 2-dose human papillomavirus (HPV) vaccination programme with the AS04-adjuvanted HPV16/18 vaccine (AS04-HPV-16/18v)HPV-6/11/16/18 vaccine (4vHPVv), in addition to cervical cancer (CC) screening, in Malaysia.
Van Minh et al., 2017[38]	Vietnam	LMIC	To perform the first local cost-effectiveness analysis of human papillomavirus vaccines in Vietnam using the Papillomavirus Rapid Interface for Modelling and Economics (PRIME).
Wahab et al., 2023[39]	Singapore	HIC	To assess if including 13-year-old boys in Singapore's national school-based HPV vaccination programme is economically beneficial from the healthcare payer's perspective.
Yamabe et al., 2014[40]	Japan	HIC	To assess the epidemiological and economic impact of quadrivalent human papillomavirus (HPV) (6/11/16/18) vaccine for females in preventing cervical cancer, cervical intraepithelial neoplasia grades 2 and 3 (CIN2/3), cervical intraepithelial

			neoplasia grade 1 (CIN1), and genital warts in Japan by using a transmission dynamic model.
Yamamoto et al., 2011[41]	Japan	HIC	To assess the cost-effectiveness of universal vaccination of 11-year-old girls against human papillomavirus (HPV) infection and increased screening coverage to prevent cervical cancer in Japan, where the coverage of Papanicolaou smears is very low.
Zhang et al., 2016[42]	China	UMIC	To assess the cost-effectiveness of 3 doses of the bivalent HPV vaccine in rural and urban settings in China
Zhou et al., 2022[43]	China	UMIC	To assess the cost-effectiveness of incorporating different HPV vaccines into immunisation programs at the Chinese national and provincial levels.
Zou et al., 2020[44]	China	UMIC	To evaluate the cost-effectiveness of the combined strategies of cervical cancer screening programmes and universal vaccination of girls (aged 9–14 years) with Cecolin in China.

Table S6. Characteristics of the cost-effectiveness of HPV vaccination studies

Authors, years of publication	Target Age	Sex of cohort	Vaccine delivery route	Type of vaccine	No of doses	Type of model	Threshold	Perspective	Time Horizon (years)	Discount rate	Sensitivity analysis	Most sensitive parameter	GDP per capita
Aljunid et al., 2016[2]	13 years	Female	NIP	N/A	2 &3	Markov	N/A	Health system	95	3%	One-way	N/A	N/A
Blakely et al., 2014[3]	12 years	Gender Neutral	NIP	4vHPV	3	Markov	GDP	Health system	98	3%	One-way	vaccine price, lesser herd immunity benefits, discount rate	NZD 40,000
Cheung et al., 2021[4]	12 years	Female	NIP	9vHPV	2 & 3	Dynamic	GDP	Health system	100	3%	One-way	Vaccine discount rate	
Cheung et al., 2023[5]	12 years	Gender Neutral	NIP	9vHPV	N/A	Dynamic	GDP	Health system	100	3%	One-way	Vaccine discount rate	HKD 382,046
Chou et al., 2022[6]	13-14 years	Female	NIP	2vHPV, 9vHPV	2	Dynamic	GDP	N/A	100	3%	One-way	ICER value	NTD 746,526
Cody et al., 2021[7]	12-16 years	Female	NIP	4vHPV, 9vHPV	2 & 3	Continuous time	WTP	Health system	100	2%	One-way	Discount rate	N/A
Connelly et al., 2015[8]	12 years	Female	NIP	2vHPV	N/A	Markov	GDP	Health system	73	5%	One-way	discount rate, the cost of vaccine, vaccine efficacy, vaccine coverage, duration of protection	USD 36,300-108,000
Dasbach et al., 2008[9]	12 years	Female	NIP	4vHPV	3	Dynamic	GDP	Health system	100	3%	One-way	duration of vaccine protection	NTD 512,000
Demarteau et al., 2012[10]	12 years	Female	NIP	2vHPV, 4vHPV	3	Markov	GDP	Health system	95	3%	Univariate and probabilistic sensitivity analysis	duration of vaccine protection	NTD 503,625
Ezat et al., 2010[11]	15 years	Female	NIP	2vHPV, 4vHPV	3	N/A	GDP	Health system and societal	10	3%	Scenario-based sensitivity analysis	N/A	N/A

Ezat et al., 2010[12]	9 to 26 years	Female	NIP	4vHPV	3	N/A	GDP	Health system and societal	10	3%	Scenario-based sensitivity analysis	N/A	RM 23,038
Germar et al., 2016[13]	13 years	Female	NIP	2vHPV, 4vHPV	2	Markov	GDP	Health system	95	3.50%	One-way and probabilistic sensitivity analysis	HPV 16/18 distribution in cervical cancer, cost of vaccine, screening-associated costs, vaccine efficacy, cost of treatment of genital warts	PHP 118,295
Jiang et al., 2019[14]	16 years	Female	N/A	9vHPV	3	PRIME model	GDP	Societal	100	3%	One-way	Discount rate	USD 25,920
Kim et al., 2008[15]	12 years	Female	N/A	N/A	3	Monte Carlo simulation model	GDP	Societal	N/A	3%	One- and two-way	achievable vaccination and screening coverage, vaccine efficacy, vaccine, and screening costs	I$2000
Konno et al., 2010[16]	12 years	Female	NIP	N/A	3	Markov	WTP	Health system	95	3%	Univariate	Discount rate, vaccination cost, HPV16/18 proportion rate	N/A
Kulasingam et al., 2007[17]	12 years	Female	NIP	4vHPV	3	Markov	N/A	Health system	73	5% base case, 3% sensitivity analyses	N/A	Duration of vaccine efficacy	N/A
Lee et al., 2011[18]	12 years	Female	NIP	2vHPV, 4vHPV	3	Markov	GDP	Health system	88	3%	One-way and two-way	vaccine effectiveness,	SGD 53,192

												vaccine coverage	
Levin et al., 2015[19]	9 years	Female	NIP	N/A	3	Monte Carlo simulation model	GDP	Health system	N/A	N/A	N/A	N/A	USD 3749
Liu PH et al., 2010[20]	12 years	Female	NIP	N/A	3	Markov	GDP	Health system	Lifetime	3%	One-way	Vaccination strategy	USD 17,082
Liu YJ et al., 2016[21]	12-55 years	Female	NIP	2vHPV	3	Markov	GDP	Health system	Lifetime	3%	Scenario analysis	N/A	125,723 CNY
Llave et al., 2022[22]	9 years	Female	NIP	2vHPV (2 type of brand), 4vHPV, 9vHPV	2	UNIVAC model	GDP	Health system & societal	Lifetime	5.33% base case analysis; 3% & 10% scenario analyses.	Univariate sensitivity analyses. Probabilistic sensitivity analyses.	Discount rate	USD 3,485
Luo et al., 2020[23]	12 years	Female	NIP	2vHPV	2	Markov	GDP	Health system	88	3% (base-case analysis); 1-5%	One-way	vaccine cost	92,100 CNY
Mahumud et al., 2019[24]	12 years	Female	NIP	9vHPV	2	PRIME	GDP	Health system and societal	20	5%	Deterministic sensitivity analysis	vaccine cost	AUD 73,267
Ma et al., 2020[25]	9 to 16 years	Female	NIP	4vHPV	N/A	Dynamic	GDP	Health system	50	3% base-case; 10-90% discount 4vHPV price scenarios	Probability and univariate	vaccine cost, vaccine coverage, screening coverage	USD 10,264
Mo et al., 2017[26]	12 years	Female	NIP	2vHPV, 4vHPV, 9vHPV	3	Markov	GDP, WTP	Societal	N/A	3%	One-way	Discount rate of effectiveness, screening coverage, discount rate of cost, utility of warts, CIN 3, age of vaccination	USD 7960

Phua et al., 2021[27]	13 years	Female	NIP	2vHPV, 4vHPV	2	Markov Chain Monte Carlo	WTP	Health system	100	3%	Probabilistic sensitivity analysis	Cross-protective vaccine efficacy	N/A
Sharma et al., 2012[28]	9 years	Female	NIP	N/A	3	Monte Carlo simulation model	GDP	Societal	Lifetime	3%	N/A	Vaccine waning	I$8100
Sharma et al., 2015[29]	9 years	Gender Neutral	NIP	4vHPV	3	Dynamic	GDP	Societal	Lifetime	3%	N/A	Coverage level	I$2800
Simms et al., 2016[30]	12-13 years	Gender Neutral	NIP	4vHPV, 9vHPV	2 & 3	Dynamic	WTP	Health system	20	5%	Probabilistic sensitivity analysis	N/A	WTP AUD 30,000
Tang et al., 2019[31]	12 years	Female	NIP	2vHPV, 4vHPV, 9vHPV	2 & 3	Markov	GDP, WTP	Health system	95	3%	Univariate and probabilistic sensitivity analysis	N/A	NTD 727,818
Tay et al., 2017[32]	11-12 years	Female	NIP	2vHPV, 4vHPV, 9vHPV	2	Dynamic	GDP	Health system	100	3%	Individual	Duration of vaccine protection	N/A
Tay et al., 2017[33]	11-12 years	Gender Neutral	NIP	4vHPV	2 & 3	Dynamic	GDP	Health system	100	3%	N/A	N/A	SGD 69 050
Tay et al., 2018[34]	12 years	Female	NIP	AS04-HPV-16/18v, 4vHPV	2	Markov	GDP	Health system	Lifetime	3%	One-way	Discount rate	SGD 70,967
Termrungruanglert et al., 2012[35]	12 years	Female	NIP	4vHPV	3	Markov	GDP	Health system	88	3%	One-way	The cost of the vaccine, the efficacy of the vaccine, and the vaccine coverage rate	135,415 THB
Termrungruanglert et al., 2021[36]	11–12 years	Female	NIP	4vHPV	2 & 3	Dynamic	GDP	Health system	100	3%	N/A	N/A	160,000 THB
Van Kriekinge et al., 2017[37]	13 years	Female	NIP	AS04-HPV-16/18v, 4vHPV	2	Markov	GDP	Health system	Lifetime	3%	One-way, two-way, probabilistic (PSA)	Discount rate	N/A

Van Minh et al., 2017[38]	11 years	Female	NIP	2vHPV, 4vHPV	3	PRIME	GDP	Health system	Lifetime	3%	Model uncertainty	Vaccine price	N/A
Wahab et al., 2023[39]	13 years	Gender Neutral	NIP	2vHPV, 9vHPV	2	PRIME	WTP	Health system	87	3%	PSA, One-way	ICER	N/A
Yamabe et al., 2014[40]	12 years, catch-up 12-24 years	Female	NIP	4vHPV	3	Dynamic	GDP	Societal	100	3%	Sensitivity analysis from prior models, Pessimistic scenario sensitivity analysis	Duration of vaccine protection	¥ 3,758,000
Yamamoto et al., 2011[41]	11 years	Female	NIP	2vHPV	3	Markov	WTP	Societal	50	3%	Two-dimensional probabilistic sensitivity analysis	Higher screening rate, vaccination strategy	N/A
Zhang et al., 2016[42]	12 years	Female	NIP	2vHPV	3	Markov	GDP	Health system	Lifetime	3%	One-way, two-way, probabilistic (PSA)	cost of vaccination, discount rate, HPV infection rate, progression rate, cost of cancer	41,908 CNY
Zhou et al., 2022[43]	9 years (2vHPV, 4vHPV); 16 years (9vHPV)	Female	NIP	2vHPV (imported and domestic), 4vHPV, 9vHPV	3	PRIME	GDP	Health system	N/A	3%	Univariate	Discount rate, vaccine efficacy, vaccine price	>USD 13,655 in 6 economically developed regions
Zou et al., 2020[44]	9-14 years	Female	NIP	2vHPV (domestic)	2	Markov	WTP	Health system	Lifetime	3%	Univariate and probabilistic sensitivity analysis	N/A	USD 10,276

Table S7. Summary of vaccine characteristics and cost-effectiveness analysis results

Authors, years of publication	Type of vaccine	Vaccine efficacy	Vaccine coverage	Vaccine per dose	Duration of vaccine protection	Herd effect	Unit of cost-effectiveness	Conclusion or recommendation	Study funder
Aljunid et al., 2016[2]	N/A	98%	100%	MYR 134	Lifetime	No	QALYs	2-doses HPV vaccination may enable to protect Malaysian women at a lower cost compared to the 3-dose scheme, avoiding the same number of cervical cancer cases and deaths	GlaxoSmithKline Pty Ltd.
Blakely et al., 2014[3]	4vHPV	99%	73% school-based; 93% school-based with opt-out permitted	$113 NZ	20 years	Yes	QALYs	New Zealand's HPV vaccination program is cost-effective and pro-equity. A school-only program with 73% coverage may be a better intervention for health gain.	Health Research Council of New Zealand
Cheung et al., 2021[4]	9vHPV	100%	70%	HKD 1390	Lifetime	Yes	QALYs	The Hong Kong vaccination strategy is cost-effective and supports a catch-up plan to the current routine 9vHPV female vaccination program.	Merck Sharp & Dohme Corp.
Cheung et al., 2023[5]	9vHPV	100%	70%	HKD 1,390	Lifetime	Yes	QALYs	Routine GNV with the 9vHPV vaccine is cost-effective and reduces HPV-related diseases, including head and neck and penile cancer.	Merck Sharp & Dohme Corp.
Chou et al., 2022[6]	2vHPV, 9vHPV	100%	70%	9vHPVv NTD 2300; 2vHPVv NTD 630	Lifetime	Yes	QALYs	A nonavalent HPV vaccine for 13-14-year-old girls is cost-effective compared to the bivalent vaccine and has additional public health and economic benefits.	Merck Sharp & Dohme LLC

Cody et al., 2021[7]	4vHPV, 9vHPV	N/A	70%	4vHPVv ¥12,000; 9vHPVv ¥20,000	Lifetime	Yes	QALYs	HPV vaccination in Japan is highly beneficial. The 9vHPV vaccination program with a catch-up for females up to age 26 is the most cost-effective approach.	Not declared
Connelly et al., 2015[8]	2vHPV	100%	80%	USD 120	Lifetime	No	QALYs	Adding HPV vaccination to Japan's cervical cancer screening program is a cost-effective way to reduce the burden of cervical cancer and related diseases.	National Health and Medical Research Council Australia
Dasbach et al., 2008[9]	4vHPV	90%	0-85%	NTD 11,800 (for 3 doses and administration)	Lifetime	No	QALYs	Vaccinating females with 4vHPV can significantly reduce genital warts, CIN, and cervical cancer, improve quality of life and survival, and be cost-effective with a temporary catch-up program.	N/A
Demarteau et al., 2012[10]	2vHPV, 4vHPV	98%	100%	NTD 3500	Lifetime	No	QALYs	In Taiwan, HPV mass vaccination with the bivalent vaccine was estimated to dominate vaccination with the quadrivalent vaccine under most assumptions.	GlaxoSmithKline Biologicals
Ezat et al., 2010[11]	2vHPV, 4vHPV	95%	95%	RM 100	Lifetime	No	QALYs	Quadrivalent vaccine is more cost-effective than the bivalent vaccine. The quadrivalent vaccine combined with Pap smear screening was more cost-effective than any method with high population coverage.	Merck, Sharp and Dome through the Public Health Specialist Association of Malaysia.

Ezat et al., 2010[12]	4vHPV	95%	95%	RM 300-400	Lifetime	No	QALYs	Vaccination prevents cancer and increases women's life expectancy and quality of life. Increasing Pap smear coverage to 70% is cost-effective. However, vaccinating young women is a more cost-effective strategy against cervical cancer, as long-term screening adherence is doubtful among Malaysian women.	Not declared
Germar et al., 2016[13]	2vHPV, 4vHPV	98%	100%	PHP 1,000	Lifetime	No	QALYs	AS04-HPV-16/18 is shown to be dominant over 4vHPV in the Philippines, with greater estimated health benefits and lower costs.	GlaxoSmithKline Biologicals S.A.
Jiang et al., 2019[14]	9vHPV	100%	100%	9vHPVv USD 610; 4vHPVv USD 375; 2vHPVv USD 273	Lifetime	No	QALYs	The 9-valent HPV vaccine is not cost-effective compared to the quadrivalent vaccine or the bivalent vaccine for young Chinese girls who have not been previously infected with HPV. However, it is marginally cost-effective when compared with no vaccination.	N/A
Kim et al., 2008[15]	N/A	100%	70%	I$2-127	Lifetime	N/A	YLS	HPV vaccination is an attractive strategy for preventing cervical cancer in Vietnam if high coverage is achieved in young girls and the cost per vaccinated girl is <$5 per dose. Screenings	Bill and Melinda Gates Foundation

								should be offered at older ages.	
Konno et al., 2010[16]	N/A	95%	50%	¥36,000	Lifetime	N/A	QALYs	The implementation of cervical cancer vaccination is predicted to be cost-effective in Japan.	N/A
Kulasingam et al., 2007[17]	4vHPV	100%	80%	AUD 115	Lifetime	Yes	QALYs	Adding the HPV vaccine to Australia's screening regimen can cost-effectively reduce cervical cancer and associated clinical interventions.	Unrestricted grant from CSL Australia
Lee et al., 2011[18]	2vHPV, 4vHPV	95%	100%	SGD 400	Lifetime	No	QALYs	HPV vaccination is a cost-effective strategy and should be considered a possible strategy to reduce the impact of HPV infection	GSK Biologicals
Levin et al., 2015[19]	N/A	N/A	70%	USD 13	Lifetime	No	YLS	HPV vaccination is cost-effective and can reduce cancer by 44% when combined with cervical cancer screening in adulthood. This holds true for all income groups if the cost of vaccination is less than USD 50 per vaccinated girl.	The Bill & Melinda Gates Foundation
Liu PH et al., 2010[20]	N/A	75%	100%	USD 121	Lifetime	No	QALYs	HPV vaccination of preadolescent girls in Taiwan would result in substantial population benefits with a favourable cost-effectiveness ratio.	Department of Health, Executive Yuan of Taiwan
Liu YJ et al., 2016[21]	2vHPV	93.20%	70%	633 CNY	Lifetime	No	QALYs	Vaccination is cost-effective for children under 23 years in rural and under 25 years in urban areas. Catch-up till	GlaxoSmithKline Biologicals

								the age of 25 is still cost-effective in both regions.	
Llave et al., 2022[22]	2vHPV (2 type of brand), 4vHPV, 9vHPV	Without cross-protection: Cervarix: 1 dose 63.4%; 2-dose70.4%; Cecolin: 1 dose: 64.0%; 2-dose 71.2%; Gardasil-4: 1-dose 62,9%; 2-dose: 69,9%; Gardasil-9: 1-dose 82,3%; 2-dose 91.4%; With cross-protection: Cervarix: 1 -dose 80.1%; Cecolin: 1 dose 64.5%; Gardasil 4: 1-dose 63.4%	Dose-1: 78.0%; Dose-2: 60,5%	Cervarix USD 10.68; Cecolin USD 7.47; Gardasil-4 USD 13.14; Gardasil-9 USD 44.64	Lifetime	No	DALYs	Administering Cecolin, Cervarix, or GARDASIL vaccines in the Philippines as a part of NIP is cost-effective compared to no vaccination.	BMBF (German Federal Ministry of Education and Research)
Luo et al., 2020[23]	2vHPV	40-100%	70%	50-1000 CNY	Lifetime	No	QALYs	The 2vHPV vaccine would be highly cost-effective at a per-dose vaccine price of CNY 500. This has implications for cervical cancer control in China and other resource-limited countries.	Medical Health Science and Technology Project of Zhejiang Province, China
Mahumud et al., 2019[24]	9vHPV	100%	86%	AUD 93	20 years	Yes	DALYs	The 9vHPV vaccine is very cost-effective and most viable in Australia for preadolescent girls.	The PhD program was funded by the University of Southern Queensland, Australia.
Ma et al., 2020[25]	4vHPV	93.3-100%	0-90%	N/A	N/A	No	DALYs	Screening adult women for cervical cancer is the most affordable way to maximise health benefits. Vaccinating schoolgirls is beneficial in the long term, but a universal HPV vaccination program requires a significant cost reduction to be cost-effective.	National Natural Science Foundation of China; Outstanding Young Scholars Funding; Xi'an Jiaotong University Basic Research and Profession Grant

Mo et al., 2017[26]	2vHPV, 4vHPV, 9vHPV	2vHPV: 96.5%; 4vHPV: 96.0%; 9vHPV: 96.0% and 96.7%	2vHPV 80.72%; 4vHPV 81.51%; 9vHPV 90.78%	2vHPVv and 4vHPVv: USD 134 ; 9vHPVv USD 149	Lifetime	No	QALYs	The HPV4/9 vaccination and screening are cost-effective in reducing HPV-related disease burden in China.	Grants from the Japan Society for the Promotion of Sciences, the National Centre for Child Health and Development, and the Chinese Natural Sciences Foundation
Phua et al., 2021[27]	2vHPV, 4vHPV	95%	91.50%	2vHPVv SGD 61.50; 9vHPVv SGD 188	Lifetime	Yes	QALYs	The 9vHPV vaccine is not a cost-effective option compared with the 2vHPV vaccine.	Did not receive any specific grant
Sharma et al., 2012[28]	N/A	100%	80%	I$ 10 -500	Lifetime	No	YLS	Low-cost preadolescent HPV vaccination followed by HPV screening five times per lifetime is an efficient strategy for Thailand.	Bill & Melinda Gates Foundation.
Sharma et al., 2015[29]	4vHPV	100% for girls; 85% for boys	0-90%	I$ 5	Lifetime	Yes	QALYs	Vaccinating boys may be cost-effective at low cost but provides little benefit over girls-only vaccination.	Bill & Melinda Gates Foundation.
Simms et al., 2016[30]	4vHPV, 9vHPV	95% for girls; 85% for boys	Three dose 4vHPV vaccine 70% girls, 65% boys	N/A	Lifetime	Yes	QALYs	9vHPV vaccine will be cost-effective compared to 4vHPV vaccine if the cost per dose is AUD 23-36 (USD 18-28)	National Health and Medical Research Council, Australia
Tang et al., 2019[31]	2vHPV, 4vHPV, 9vHPV	AS04-HPV-16/18v 93.2%; 4vHPV 43.0%; 9vHPV 79.6%	N/A	AS04-HPV-16/18v: NTD 1,800; 4vHPVv: NTD 1,800 and 9vHPVv: NTD 2,500	Lifetime	No	QALYs	Vaccinating Taiwanese girls with 2D-AS04-HPV-16/18v in addition to screening is cost-effective compared to screening alone and the dominant option compared with 2D-4vHPVv+screening and 2D/3D-9vHPVv+screening	GlaxoSmithKline Biologicals SA

Tay et al., 2017[32]	2vHPV, 4vHPV, 9vHPV	HPV types: 16 76%; 18 96.3%; 31/33/45/52/58 nonavalent 76%	80%	2vHPVv SGD 90; 4vHPVv SGD 12; 9vHPVv SGD 180	Lifetime	N/A	QALYs	Universal two-dose nonavalent HPV vaccination for 11- to 12-year-old adolescent women is very cost-effective in Singapore.	N/A
Tay et al., 2017[33]	4vHPV	CIN 95.2%, genital warts 98.9%	80% 11-12 years girls and boys; 50% coverage for 13-17 years girls	SGD 160	Lifetime	N/A	QALYs	School-based quadrivalent HPV vaccination offers clinical and economic benefits and is cost-effective in Singapore.	N/A
Tay et al., 2018[34]	AS04-HPV-16/18v, 4vHPV	98%	90%	SGD 90	Lifetime	No	QALYs	AS04-HPV-16/18v is the most cost-effective choice for reducing the burden of cervical cancer through universal mass vaccination for 12-year-old girls in Singapore.	GlaxoSmithKline Biologicals SA
Termrungruanglert et al., 2012[35]	4vHPV	97%	80-100%	2063 THB	LIfetime	No	QALYs	The prophylactic quadrivalent vaccine was likely to be cost-effective in Thailand.	N/A
Termrungruanglert et al., 2021[36]	4vHPV	N/A	95%	500 THB	Lifetime	Yes	QALYs	Implementing the routine 4vHPV vaccination, either alone or plus the catch-up vaccination, was cost-effective compared to the cervical cancer screening only.	MSD (Thailand) Ltd
Van Kriekinge et al., 2017[37]	AS04-HPV-16/18v, 4vHPV	98%	100%	N/A	Lifetime	No	QALYs	In Malaysia, the use of AS04-HPV-16/18v, in addition to screening, was modelled to be dominant over 4vHPVv.	GlaxoSmithKline Biologicals S.A.
Van Minh et al., 2017[38]	2vHPV, 4vHPV	N/A	95%	GAVI USD 4.55; Cervarix USD 35.60; Gardasil USD 55.80	N/A	No	DALYs	HPV vaccine introduction appears to be economically attractive only if Vietnam can procure the vaccine at Gavi prices.	WHO and Gavi, the Vaccine Alliance

Wahab et al., 2023[39]	2vHPV, 9vHPV	100%	80%	2vHPVv SGD 61.5; 9vHPVv SGD 188	Lifetime	No	QALYs	Switching to a gender-neutral vaccination program with a bivalent HPV vaccine may be cost-effective in Singapore, but not if the program shifts to a nonavalent HPV vaccine at current prices.	N/A
Yamabe et al., 2014[40]	4vHPV	90%	80%	¥36,000	Lifetime	N/A	QALYs	A quadrivalent HPV vaccination program for females is cost-effective in reducing the incidence of cervical cancer, CIN, and genital warts in Japan.	MSD K.K., Tokyo, Japan, and Merck, Nort Wales, PA, USA
Yamamoto et al., 2011[41]	2vHPV	N/A	N/A	¥58,000	Lifetime	N/A	QALYs	The introduction of HPV vaccination in Japan is as cost-effective as in other countries. It is more cost-effective to increase the coverage of the Papanicolaou smear along with the universal administration of the HPV vaccine.	Health and Labour Sciences Research Grants from the Japanese Ministry of Health, Labour and Welfare and a Research accomplishment cooperation system grant for doctoral courses at the University of Tokyo.
Zhang et al., 2016[42]	2vHPV	93.20%	70%	247 CNY	Lifetime	No	QALYs	HPV vaccination is crucial in areas with high cervical cancer incidence and limited screening activities. The cost and discounts available play an essential role in its cost-effectiveness. However, vaccine costs must be reduced to make vaccines affordable in poverty-	GlaxoSmithKline Biologicals SA; National Natural Science Foundation of China and NIH Fogarty International Center Grant

								stricken areas with a high disease burden.	
Zhou et al., 2022[43]	2vHPV (imported and domestic), 4vHPV, 9vHPV	100%	80%	Domestic 2vHPVv USD 47.7; Imported 2vHPVv USD 84.10; 4vHPVv USD 115.70; 9vHPVv USD 188.2	N/A	No	DALYs	HPV vaccination is cost-effective at a national level and in most provinces, with higher population provinces having more prevented cases, deaths, and DALYs.	National Natural Science Fund
Zou et al., 2020[44]	2vHPV (domestic)	94%	70%	USD 47.40	Lifetime	No	QALYs	CareHPV screening every five years plus vaccination is the most cost-effective way to prevent cervical cancer in China. Lower domestic HPV vaccine prices are needed for an economical vaccination program.	National Natural Science Foundation of China

References

1. Husereau, D.; Drummond, M.; Augustovski, F.; de Bekker-Grob, E.; Briggs, A.H.; Carswell, C.; Caulley, L.; Chaiyakunapruk, N.; Greenberg, D.; Loder, E.; et al. Consolidated Health Economic Evaluation Reporting Standards 2022 (CHEERS 2022) Statement: Updated Reporting Guidance for Health Economic Evaluations. *Value Health* **2022**, *25*, 3-9, doi:10.1016/j.jval.2021.11.1351.
2. Aljunid, S.; Maimaiti, N.; Nur, A.M.; Noor, M.R.; Puteh, S.E. Cost-effectiveness of HPV vaccination regime: comparing twice versus thrice vaccinations dose regime among adolescent girls in Malaysia. *BMC public health* **2016**, *16*, 71, doi:10.1186/s12889-016-2754-1.
3. Blakely, T.; Kvizhinadze, G.; Karvonen, T.; Pearson, A.L.; Smith, M.; Wilson, N. Cost-effectiveness and equity impacts of three HPV vaccination programmes for school-aged girls in New Zealand. *Vaccine* **2014**, *32*, 2645-2656, doi:10.1016/j.vaccine.2014.02.071.
4. Cheung, T.H.; Cheng, S.S.Y.; Hsu, D.C.; Wong, Q.W.L.; Pavelyev, A.; Walia, A.; Saxena, K.; Prabhu, V.S. The impact and cost-effectiveness of 9-valent human papillomavirus vaccine in adolescent females in Hong Kong. *Cost Effectiveness and Resource Allocation* **2021**, *19*, doi:10.1186/s12962-021-00328-x.
5. Cheung, T.H.; Cheng, S.S.Y.; Hsu, D.; Wing-Lei Wong, Q.; Pavelyev, A.; Sukarom, I.; Saxena, K. Health impact and cost-effectiveness of implementing gender-neutral vaccination with the 9-valent HPV vaccine in Hong Kong. *Hum Vaccin Immunother* **2023**, *19*, 2184605, doi:10.1080/21645515.2023.2184605.
6. Chou, H.H.; Chang, S.C.; Sukarom, I.; Saxena, K.; Pavelyev, A.; Wu, Y.H.; Chang, C.J. The Clinical and Economic Impact of a Nonavalent Versus Bivalent Human Papillomavirus National Vaccination Program in Taiwan. *Value in Health Regional Issues* **2022**, *32*, 79-87, doi:10.1016/j.vhri.2022.06.006.
7. Cody, P.; Tobe, K.; Abe, M.; Elbasha, E.H. Public health impact and cost effectiveness of routine and catch-up vaccination of girls and women with a nine-valent HPV vaccine in Japan: a model-based study. *BMC Infectious Diseases* **2021**, *21*, doi:10.1186/s12879-020-05632-0.
8. Connelly, L.B.; Le, H.N.D. Cost-effectiveness of a bivalent human papillomavirus vaccination program in Japan. *Sexual Health* **2015**, *12*, 520-531, doi:10.1071/SH14241.
9. Dasbach, E.J.; Insinga, R.P.; Yang, Y.C.; Pwu, R.F.; Lac, C.; Elbasha, E.H. The cost-effectiveness of a quadrivalent human papillomavirus vaccine in Taiwan. *Asian Pacific Journal of Cancer Prevention* **2008**, *9*, 459-466.
10. Demarteau, N.; Tang, C.H.; Chen, H.C.; Chen, C.J.; Van Kriekinge, G. Cost-effectiveness analysis of the bivalent compared with the quadrivalent human papillomavirus vaccines in Taiwan. *Value in Health* **2012**, *15*, 622-631, doi:10.1016/j.jval.2012.02.012.
11. Ezat, S.W.P.; Aljunid, S. Comparative cost-effectiveness of HPV vaccines in the prevention of cervical cancer in Malaysia. *Asian Pacific Journal of Cancer Prevention* **2010**, *11*, 943-951.
12. Ezat, W.P.; Aljunid, S. Cost-effectiveness of HPV vaccination in the prevention of cervical cancer in Malaysia. *Asian Pacific journal of cancer prevention : APJCP* **2010**, *11*, 79-90.
13. Germar, M.J.; Purugganan, C.; Bernardino, M.S.; Cuenca, B.; Chen, Y.C.; Li, X.; Van Kriekinge, G.; Lee, I.H. Cost-effectiveness analysis of AS04-adjuvanted human papillomavirus 16/18 vaccine compared with human papillomavirus 6/11/16/18 vaccine in the Philippines, with the new 2-dose schedule. *Human Vaccines and Immunotherapeutics* **2017**, *13*, 1158-1166, doi:10.1080/21645515.2016.1269991.
14. Jiang, Y.; Ni, W.; Wu, J. Cost-effectiveness and value-based prices of the 9-valent human papillomavirus vaccine for the prevention of cervical cancer in China: An economic modelling analysis. *BMJ Open* **2019**, *9*, doi:10.1136/bmjopen-2019-031186.

15. Kim, J.J.; Kobus, K.E.; Diaz, M.; O'Shea, M.; Van Minh, H.; Goldie, S.J. Exploring the cost-effectiveness of HPV vaccination in Vietnam: Insights for evidence-based cervical cancer prevention policy. *Vaccine* **2008**, *26*, 4015-4024, doi:10.1016/j.vaccine.2008.05.038.
16. Konno, R.; Sasagawa, T.; Fukuda, T.; Van Kriekinge, G.; Demarteau, N. Cost-effectiveness analysis of prophylactic cervical cancer vaccination in Japanese women. *International Journal of Gynecological Cancer* **2010**, *20*, 385-392, doi:10.1111/IGC.0b013e3181d189b8.
17. Kulasingam, S.; Connelly, L.; Conway, E.; Hocking, J.S.; Myers, E.; Regan, D.G.; Roder, D.; Ross, J.; Wain, G. A cost-effectiveness analysis of adding a human papillomavirus vaccine to the Australian National Cervical Cancer Screening Program. *Sexual Health* **2007**, *4*, 165-175, doi:10.1071/SH07043.
18. Lee, V.J.; Tay, S.K.; Teoh, Y.L.; Tok, M.Y. Cost-effectiveness of different human papillomavirus vaccines in Singapore. *BMC public health* **2011**, *11*, 203, doi:10.1186/1471-2458-11-203.
19. Levin, C.E.; Sharma, M.; Olson, Z.; Verguet, S.; Shi, J.-F.; Wang, S.-M.; Qiao, Y.-L.; Jamison, D.T.; Kim, J.J. An extended cost-effectiveness analysis of publicly financed HPV vaccination to prevent cervical cancer in China. *Vaccine* **2015**, *33*, 2830-2841, doi:https://doi.org/10.1016/j.vaccine.2015.02.052.
20. Liu, P.H.; Hu, F.C.; Lee, P.I.; Chow, S.N.; Huang, C.W.; Wang, J.D. Cost-effectiveness of human papillomavirus vaccination for prevention of cervical cancer in Taiwan. *BMC Health Serv Res* **2010**, *10*, 11, doi:10.1186/1472-6963-10-11.
21. Liu, Y.J.; Zhang, Q.; Hu, S.Y.; Zhao, F.H. Effect of vaccination age on cost-effectiveness of human papillomavirus vaccination against cervical cancer in China. *BMC Cancer* **2016**, *16*, doi:10.1186/s12885-016-2207-3.
22. Llave, C.L.; Uy, M.E.V.; Lam, H.Y.; Aldaba, J.G.; Yacapin, C.C.; Miranda, M.B.; Valverde, H.A.; Silva, W.T.; Nawaz, S.; Slavkovsky, R.C.; et al. The cost-effectiveness of human papillomavirus vaccination in the Philippines. *Vaccine* **2022**, *40*, 3802-3811, doi:10.1016/j.vaccine.2022.05.025.
23. Luo, Y.; He, H.; Tang, X.; Wang, S.; Zhang, J.; Wu, T.; Chen, Z. Cost-effectiveness of 2-dose human papillomavirus vaccination for 12-year-old girls in Zhejiang Province: implications for China's expanded program on immunization. *Human Vaccines and Immunotherapeutics* **2020**, *16*, 1623-1629, doi:10.1080/21645515.2019.1711299.
24. Mahumud, R.A.; Alam, K.; Dunn, J.; Gow, J. The cost-effectiveness of controlling cervical cancer using a new 9-valent human papillomavirus vaccine among school-aged girls in Australia. *PLoS ONE* **2019**, *14*, doi:10.1371/journal.pone.0223658.
25. Ma, X.; Harripersaud, K.; Smith, K.; Fairley, C.K.; Zou, H.; Zou, Z.; Wang, Y.; Zhuang, G.; Zhang, L. Modeling the epidemiological impact and cost-effectiveness of a combined schoolgirl HPV vaccination and cervical cancer screening program among Chinese women. *Human Vaccines and Immunotherapeutics* **2021**, *17*, 1073-1082, doi:10.1080/21645515.2020.1832835.
26. Mo, X.; Gai Tobe, R.; Wang, L.; Liu, X.; Wu, B.; Luo, H.; Nagata, C.; Mori, R.; Nakayama, T. Cost-effectiveness analysis of different types of human papillomavirus vaccination combined with a cervical cancer screening program in mainland China. *BMC Infectious Diseases* **2017**, *17*, doi:10.1186/s12879-017-2592-5.
27. Phua, L.C.; Choi, H.C.W.; Wu, J.; Jit, M.; Low, J.; Ng, K.; Pearce, F.; Hall, C.; Abdul Aziz, M.I. Cost-effectiveness analysis of the nonavalent human papillomavirus vaccine for the prevention of cervical cancer in Singapore. *Vaccine* **2021**, *39*, 2255-2263, doi:10.1016/j.vaccine.2021.03.040.
28. Sharma, M.; Ortendahl, J.; Van Der Ham, E.; Sy, S.; Kim, J.J. Cost-effectiveness of human papillomavirus vaccination and cervical cancer screening in Thailand. *BJOG: An International Journal of Obstetrics and Gynaecology* **2012**, *119*, 166-176, doi:10.1111/j.1471-0528.2011.02974.x.

29. Sharma, M.; Sy, S.; Kim, J.J. The value of male human papillomavirus vaccination in preventing cervical cancer and genital warts in a low-resource setting. *BJOG* **2016**, *123*, 917-926, doi:10.1111/1471-0528.13503.
30. Simms, K.T.; Laprise, J.F.; Smith, M.A.; Lew, J.B.; Caruana, M.; Brisson, M.; Canfell, K. Cost-effectiveness of the next generation nonavalent human papillomavirus vaccine in the context of primary human papillomavirus screening in Australia: a comparative modelling analysis. *The Lancet Public Health* **2016**, *1*, e66-e75, doi:10.1016/S2468-2667(16)30019-6.
31. Tang, C.H.; Cheng, W.F.; Jiang, J.H.; You, S.L.; Huang, L.W.; Hsieh, J.Y.; Mukherjee, P.; Van Kriekinge, G.; Lee, C. Cost-Effectiveness Analysis of Human Papillomavirus Vaccination in Adolescent Girls in Taiwan. *Asian Pacific journal of cancer prevention : APJCP* **2019**, *20*, 1377-1387, doi:10.31557/apjcp.2019.20.5.1377.
32. Tay, S.K.; Hsu, T.Y.; Pavelyev, A.; Walia, A.; Kulkarni, A.S. Clinical and economic impact of school-based nonavalent human papillomavirus vaccine on women in Singapore: a transmission dynamic mathematical model analysis. *BJOG: An International Journal of Obstetrics and Gynaecology* **2018**, *125*, 478-486, doi:10.1111/1471-0528.15106.
33. Tay, S.K.; Hsu, T.Y.; Shcheprov, A.; Walia, A.; Kulkarni, A.S. The clinical and economic benefits of school-based quadrivalent HPV vaccination in Singapore. *International Journal of Gynecology and Obstetrics* **2017**, *137*, 129-137, doi:10.1002/ijgo.12126.
34. Tay, S.K.; Lee, B.W.; Sohn, W.Y.; Lee, I.H.; Mathur, G.; Sanicas, M.; Van Kriekinge, G. Cost-effectiveness of two-dose human papillomavirus vaccination in Singapore. *Singapore Medical Journal* **2018**, *59*, 370-382, doi:10.11622/smedj.2017085.
35. Termrungruanglert, W.; Havanond, P.; Khemapech, N.; Lertmaharit, S.; Pongpanich, S.; Khorprasert, C.; Taneepanichskul, S. Cost and effectiveness evaluation of prophylactic HPV vaccine in developing countries. *Value in Health* **2012**, *15*, S29-S34, doi:10.1016/j.jval.2011.11.007.
36. Termrungruanglert, W.; Khemapech, N.; Vasuratna, A.; Havanond, P.; Deebukkham, P.; Kulkarni, A.S.; Pavelyev, A. The epidemiologic and economic impact of a quadrivalent human papillomavirus vaccine in Thailand. *PLoS ONE* **2021**, *16*, doi:10.1371/journal.pone.0245894.
37. Van Kriekinge, G.; Sohn, W.Y.; Aljunid, S.M.; Soon, R.; Yong, C.M.; Chen, J.; Lee, I.H. Comparative Cost-Effectiveness Analysis of Two Different Two-Dose Human Papillomavirus Vaccines in Malaysia. *Asian Pac J Cancer Prev* **2018**, *19*, 933-940, doi:10.22034/APJCP.2018.19.4.933.
38. Van Minh, H.; My, N.T.T.; Jit, M. Cervical cancer treatment costs and cost-effectiveness analysis of human papillomavirus vaccination in Vietnam: a PRIME modeling study. *BMC health services research* **2017**, *17*, 353, doi:10.1186/s12913-017-2297-x.
39. Wahab, M.T.; Tan, R.K.J.; Cook, A.R.; Prem, K. Impact of including boys in the national school-based human papillomavirus vaccination programme in Singapore: A modelling-based cost-effectiveness analysis. *Vaccine* **2023**, *41*, 1934-1942, doi:10.1016/j.vaccine.2023.02.025.
40. Yamabe, K.; Singhal, P.K.; Abe, M.; Dasbach, E.J.; Elbasha, E.H. The cost-effectiveness analysis of a quadrivalent human papillomavirus vaccine (6/11/16/18) for females in Japan. *Value in Health Regional Issues* **2013**, *2*, 92-97, doi:10.1016/j.vhri.2013.02.001.
41. Yamamoto, N.; Mori, R.; Jacklin, P.; Osuga, Y.; Kawana, K.; Shibuya, K.; Taketani, Y. Introducing HPV vaccine and scaling up screening procedures to prevent deaths from cervical cancer in Japan: A cost-effectiveness analysis. *BJOG: An International Journal of Obstetrics and Gynaecology* **2012**, *119*, 177-186, doi:10.1111/j.1471-0528.2011.03036.x.
42. Zhang, Q.; Liu, Y.J.; Hu, S.Y.; Zhao, F.H. Estimating long-term clinical effectiveness and cost-effectiveness of HPV 16/18 vaccine in China. *BMC Cancer* **2016**, *16*, doi:10.1186/s12885-016-2893-x.
43. Zhou, L.; Gu, B.; Wang, J.; Liu, G.; Zhang, X. Human papillomavirus vaccination at the national and provincial levels in China: a cost-effectiveness analysis using the PRIME model. *BMC public health* **2022**, *22*, 777, doi:10.1186/s12889-022-13056-5.

44. Zou, Z.; Fairley, C.K.; Ong, J.J.; Hocking, J.; Canfell, K.; Ma, X.; Chow, E.P.F.; Xu, X.; Zhang, L.; Zhuang, G. Domestic HPV vaccine price and economic returns for cervical cancer prevention in China: a cost-effectiveness analysis. *The Lancet Global Health* **2020**, *8*, e1335-e1344, doi:10.1016/S2214-109X(20)30277-1.
